# Chinese Herbal Medicines for the Treatment of Type A H1N1 Influenza: A Systematic Review of Randomized Controlled Trials

**DOI:** 10.1371/journal.pone.0028093

**Published:** 2011-12-02

**Authors:** Wei Chen, Chi Eung Danforn Lim, Hong-Jun Kang, Jianping Liu

**Affiliations:** 1 Centre For Evidence-Based Chinese Medicine, Beijing University of Chinese Medicine, Beijing, China; 2 Faculty of Medicine, South Western Sydney Clinical School, University of New South Wales, Sydney, Australia; 3 Intensive Care Unit, Chinese PLA General Hospital, Beijing, China; 4 NAFKAM, University of Tromso, Tromso, Norway; University of Hong Kong, Hong Kong

## Abstract

**Background:**

Chinese herbs are thought to be effective for type A H1N1 influenza. Series of Chinese herbs have been authorized recommended by the Chinese government, and until now a number of clinical trials of Chinese herbs for H1N1 influenza have been conducted. However, there is no critically appraised evidence such as systematic reviews or meta-analyses on potential benefits and harms of medicinal herbs for H1N1 influenza to justify their clinical use and their recommendation.

**Methods and Findings:**

CENTRAL, MEDLINE, EMBASE, CBM, CNKI, VIP, China Important Conference Papers Database, China Dissertation Database, and online clinical trial registry websites were searched for published and unpublished randomized controlled trials (RCTs) of Chinese herbs for H1N1 influenza till 31 August, 2011. A total of 26 RCTs were identified and reviewed. Most of the RCTs were of high risk of bias with flawed study design and poor methodological quality. The combination of several Chinese herbal medicines with or without oseltamivir demonstrated positive effect on fever resolution, relief of symptoms, and global effectiveness rate compared to oseltamivir alone. However, only one herbal medicine showed positive effect on viral shedding. Most of the trials did not report adverse events, and the safety of herbal medicines is still uncertain.

**Conclusions:**

Some Chinese herbal medicines demonstrated potential positive effect for 2009 type A H1N1 influenza; however, due to the lack of placebo controlled trial and lack of repeated test of the intervention, we could not draw confirmative conclusions on the beneficial effect of Chinese herbs for H1N1 influenza. More rigorous trials are warranted to support their clinical use.

## Introduction

The influenza virus, known to be a circulating pathogen in the human population since the 16th century, is notable for its unique ability to cause recurrent epidemics and global pandemics. The ability of this virus to undergo genetic reassortments causes unpredictable changes in its antigens and the consequent immune response leads to recurrent epidemics of febrile respiratory disease every 1–3 years. In the 20th, three influenza pandemics occurred and killed tens of millions of people, with each of these pandemics being caused by the appearance of a new strain of the virus in humans [1]. In April 2009 a novel flu strain evolved that combined genes from human, pig, and bird flu, initially dubbed ‘swine flu’ and also known as influenza A/H1N1, emerged in Mexico, the United States, and several other nations. The World Health Organization officially declared the outbreak to be a pandemic on June 11, 2009 [2].

Presently, two classes of antiviral drugs have been approved by the US Food and Drug Administration (FDA) in treating or preventing influenza virus infections: M2 ion channel blockers and neuraminidase inhibitors (NAIs). The M2 blockers, amantidine and rimantidine, are effective against influenza A viruses, but not influenza B viruses, which lack the M2 protein. However, use of the M2 blockers has been associated with the rapid emergence of drug-resistance mutations of the M2 protein among human influenza A viruses of H3N2 subtype and H1N1 subtypes circulating in certain geographic areas [3]. Two NAIs, oseltamivir (Tamiflu) and zanamivir (Relenza) are approved by US FDA for use against type A and type B influenza infections. The NAIs target the active site of the NA protein, inhibiting its sialidase activity that is essential for virus release. Most of the influenza virus strains are sensitive to oseltamivir. However, it has been thought that the development of drug resistance may limit the clinical utility of the drug in the future [4].

Chinese herbs, which are the most important component of Traditional Chinese medicine (TCM), are widely used in China. Due to the limitation of healthcare resources and high cost of antiviral drugs, Chinese herbs have been recommended for preventing and treating influenza in China, especially for poor regions. In October 2009, the Ministry of Health of China issued ‘Guidelines for Management of Pandemic (H1N1) 2009 Influenza’, and recommended series of Chinese herbs for the treatment of type A H1N1 influenza, including herbal products extracted from natural herbs, Chinese patent medicine (including herbal injection), and principles for individually prescribed herbal decoction [5].

Until now, a number of clinical trials of Chinese herbs for H1N1 influenza have been conducted and reported with positive findings. However, there is no critically appraised evidence such as systematic reviews or meta-analyses on potential benefits and harms of medicinal herbs for H1N1 influenza to justify their clinical use and their recommendation.

## Methods

The supporting PRISMA checklist is available as supporting information; see Checklist S1.

### Search strategy and study selection

Literature searches were conducted in the Cochrane Central Register of Controlled Trials (CENTRAL) in the Cochrane Library (January, 2011), MEDLINE, EMBASE, Chinese BioMedical Literature Database (CBM), Chinese National Knowledge Infrastructure (CNKI), Chinese Scientific Journal Database (VIP), China's Important Conference Papers Database, and China's Dissertation Database from their inception to 31 August, 2011. Ongoing registered clinical trials were searched in the website of Chinese clinical trial registry (http://www.chictr.org/) and international clinical trial registry by U.S. national institutes of health (http://clinicaltrials.gov/). The following search terms were used individually or combined: ‘influenza’, ‘H1N1 influenza’, ‘Chinese Traditional’, ‘Chinese Herbal’, ‘Oriental Traditional’, ‘herb’, ‘herbal medicine’, ‘clinical trial’, and ‘randomized controlled trial’.

Two authors conducted the literature searching (WC, CEDL), study selection (WC, CEDL), and data extraction (WC, HJK) independently. The extracted data included authors and title of study, year of publication, study size, age and sex of the participants, details of methodological information, name and component of Chinese herbs, treatment process, details of the control interventions, outcomes (for example, total effective rate), and adverse effects for each study. Disagreement was resolved by discussion and reached consensus through a third party (JPL).

### Inclusion criteria

Parallel randomized controlled trials (RCTs) of Chinese herbs compared with no treatment, symptomatic treatment, placebo, or antivirus for H1N1 patients were included. Combined therapy of Chinese herbs and other interventions compared with other interventions in RCTs was also included. Chinese herbs included herbal products extracted from natural herbs, Chinese patent medicine, or individually prescribed herbal formula. The primary outcome measures were duration of fever (the average time for fever to clear), duration of flu-like symptoms (the average time for flu-like symptoms to disappear), and global effectiveness rate (defined as a three-class measurement including ‘cure’, ‘effective’, and ‘ineffective’ according to the degree of overall symptom improvement). The secondary outcome measures were hospital stay (number of days in hospital), viral shedding, and adverse events. Multiple publications reporting the same groups of participants were excluded. There was no limitation on language and publication type.

### Trial quality assessment

Two authors (WC, CEDL) evaluated the quality of included trials. The quality of included trials were assessed by using the ‘risk of bias’ assessment tool according to the ‘Cochrane Handbook of Systematic Reviews of Interventions’ (Chapter 8.5) to address the following five criteria [6]: sequence generation, allocation concealment, blinding, incomplete outcome data, and selective outcome reporting. The quality of all the included trials was categorized to low/unclear/high risk of bias. Trials which met all criteria were categorized to low risk of bias, trials which met none of the criteria were categorized to high risk of bias, and other trials were categorized to unclear risk of bias if insufficient information acquired to make judgment.

### Data analysis

Data were summarized using relative risk (RR) with 95% confidence intervals (CI) for binary outcomes or mean difference (MD) with 95% CI for continuous outcomes. Revman 5.0.17 software was used for data analyses. Meta-analysis was used if the trials had a good homogeneity on study design, participants, interventions, control, and outcome measures, which assessed by examining I^2^ (a quantity that describes approximately the proportion of variation in point estimates due to heterogeneity rather than sampling error). Publication bias would explored by funnel plot analysis if sufficient studies were found. If we had identified a sufficient number of randomized trials, we had planned to perform sensitivity analyses to explore the influence of trial quality on effect estimates. The quality components of methodology included adequacy of generation of allocation sequence, concealment of allocation, double blinding, and the use of intention -to -treat (yes or no).

## Results

### Description of studies

**Figure 1:**
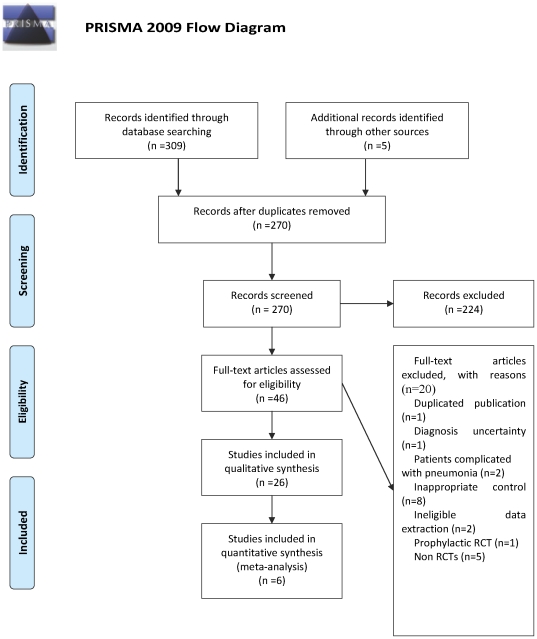
PRISMA 2009 Flow Diagram.

A flow chart depicted the search process and study selection (Fig. 1). After primary searches from the seven databases, 270 citations were screened. After reading the titles and abstracts, a majority of them was excluded. Full text of 46 papers were retrieved, and finally 26 RCTs were included [7]–[32] including two three-armed RCTs [16], [31] and four four-armed RCTs [17], [21], [24], [27]. All the RCTs were conducted in China and published in Chinese, except one published in English [24]. Twenty papers were excluded [33]–[52], and the reasons for excluded studies were listed in Table S3.

The search for ongoing registered trials identified four trials: three RCTs from the website of Chinese clinical trial registry: No. ChiCTR-TRC-10000828 investigating herbal anti-virus liquid for H1N1 influenza A; No. ChiCTR-TRC-10000814 investigating Reduning herbal injection for H1N1 influenza A; and ChiCTR-TRC-10000779 investigating TCM treatment for H1N1 influenza. All the three RCTs were either under participant recruitment or not initiated and thus the data were not available. One RCT was identified from www.clinicaltrials.gov: NCT01053533 investigating Chinese herbal medicines (details not provided) plus western medicine for H1N1 influenza A. This trial was under recruitment and data were not available.

The characteristics of included trials were listed in Table S1. A total of 2946 participants with H1N1 influenza were involved with the average number of 113 per trial, ranging from 43 to 410. All participants got influenza during the H1N1 influenza epidemic year of 2009 and were virologically confirmed as having 2009-H1N1 influenza according to Chinese Center For Disease Control And Prevention (CDC). Seven trials [7], [8], [10], [14], [18], [28], [32] provide information on patients' syndrome differentiation (Bianzheng, TCM diagnosis) as the basis for individualized herbal preparations. A total of 27 different Chinese herbs were investigated. The Chinese herbs investigated included Chinese patent medicine including single herb extract [9], [10], [11], [16], [23], [25], [29], and individually prescribed herbal formula [8], [12]–[15], [18]–[20], [22], [24], [26]–[28], [30], [32], in some trials [7], [17], [21], [31], mixed herbal medicines including multiple Chinese herbal prescription or Chinese patent medicines were tested. The specific compositions of these Chinese herbs varied as shown in Table S4. Only one trial reported the quality standard of the herbal preparations [24]. The total treatment duration ranged from 3 to 14 days. The controls included symptomatic treatment [8], [21], placebo [16], [27], and oseltamivir [7], [9]–[15], [17]–[20], [22], [23], [25]–[32]. The reported outcome measures included duration of fever, duration of flu-like symptoms, global effectiveness rate, hospital stay, viral response, and adverse events.

### Methodological quality

The majority of the included trials were assessed to be of general poor methodological quality according to the predefined quality assessment criteria. The randomized allocation of participants was mentioned in all trials; however, only 6 trials stated the methods for sequence generation including random number table [8], [16], [21], [24], drawing [31], and computer software [7]. However, insufficient information was provided to judge whether or not it was conducted properly. Allocation concealment was only mentioned in one RCT [24]. Double-blind was not mentioned in all trials. Single blind was mentioned in one trial [7] without providing further information as to who was blinded. However, as the testing Chinese herbs and controlled drug were in different forms (liquid versus tablet), neither the participants nor the investigators were likely to be blinded. Only two trials reported drop-out or withdraw [7], [24], however the trial did not intend to analyze the cause, and no trials used intention-to-treat analysis. Only one trial [24] had a pre-trial estimation of sample size, which indicated the lack of statistical power to ensure appropriate estimation of the therapeutic effect. Selective reporting was generally unclear in the RCTs due to the inaccessibility to the trial protocol.

### Effect estimates

All the trials claimed positive effect favoring Chinese herbs though some of the trials turned out to be negative when analyzed by standard statistical techniques using risk ratios or mean differences. The effect estimates of Chinese herbs were shown in the Table S2.

#### 1. Duration of fever

A total of seventeen trials [7]–[9], [12], [13], [15]–[17], [19]–[21], [23], [24], [27], [28], [29], [31], [32] reported the effect of Chinese herbs individually or in combination with oseltamivir on defervescence. Among them, eight trials demonstrated better effect favoring Chinese herbs: Fanggan Granule [8] mildly shortened fever duration than symptomatic treatment; Bingyanqing formula Ten [20] and Chinese herbal formula [17] had shorter fever duration than oseltamivir; the combination of Maxingshigan-yinqiaosan and oseltamivir had more proportion of patients who became afebrile than no intervention [24], Maxingshigan decoction combined with oseltamivir had shorter fever duration than placebo of oseltamivir [27]; Maxingshigan decoction combined with placebo of oseltamivir had shorter fever duration than placebo of oseltamivir [27]; self-prescribed herbal medicines had shorter fever duration than oseltamivir [28]; the combination of Gegen Granule [32] and oseltamivir, Tanreqing Injection [13], [19] and oseltamivir, Chinese herbal formula [17] and oseltamivir had mildly shorter fever duration than oseltamivir alone.

The forest plot of comparison of CHM versus oseltamivir for the outcome of duration of fever was shown in the Figure S1.

#### 2. Duration of flu-like symptoms

Nine trials [7]–[9], [14], [16], [23], [27], [29], [31] reported the effect of Chinese herbs individually or in combination with oseltamivir on the duration of flu-like symptoms. Among them, two trials demonstrated better effect favoring Chinese herbs: Qingwen Tuire Decoction combined with oseltamivir shortened the duration of flu-like symptoms compared to oseltamivir alone [29], and Fanggan Granule had shorter duration of flu-like symptoms compared to symptomatic treatment [8].

#### 3. Global effectiveness rate

Eleven trials [8], [10], [11], [13], [17], [18], [22], [25]–[28] reported the effect of Chinese herbs individually or in combination with oseltamivir on the global effectiveness rate. Among them, six trials demonstrated better effect favoring Chinese herbs: Qingfei Jiedu Decoction [10] showed better effect compared to oseltamivir, the combinations of oseltamivir and Tanreqing Injection [11], [13], oseltamivir and Xiyanping Injection [22] had better effect compared to oseltamivir alone, Qingjie Huashi decoction showed better effect compared to oseltamivir [25], and the combination of Reduning Injection [26] and ribavirin plus oseltamivir had better effect compared to ribavirin plus oseltamivir.

#### 4. Hospital stay

Six trials [7], [14], [16], [17], [19], [27] reported the effect of Chinese herbs individually or in combination with oseltamivir on hospital stay. Among them, two trials demonstrated better effect favoring Chinese herbs: one herbal formula showed shorter hospital stay compared to oseltamivir [17], and Tanreqing Injection combined with oseltamivir had shorter hospital stay compared to oseltamivir alone [19].

#### 5. Viral shedding

Ten trials [9], [12], [14], [15], [17], [19], [21], [28], [30], [31] reported the effect of herbal medicines individually or in combination with oseltamivir on viral shedding. Among them, one self-prescribed herbal medicines showed better effect compared with oseltamivir [28].

#### 6. Adverse events

As shown in Table S2, the outcome of adverse events were reported in fifteen trials [7], [8], [10], [12], [14]–[18], [22], [24], [25], [27], [30], [31]. Among them, no adverse events were found in three trial [12], [14], [15], [31]. Six trials reported adverse events in Chinese herbs group, which included diarrhea [7], [8], [16], arrhythmia [8], mild nausea [17], [24], vomit [24], mild upper abdominal discomfort [17], and lung infection [18]. In the trial of Tang 2010 [22], two cases of wheal were identified, but did not report in which group it occurred.

We conducted funnel plots to investigate the publication bias (see Figure S2). It demonstrated asymmetrical funnel plots, suggesting potential publication bias.

## Discussion

In this review, several Chinese herbal medicines demonstrated potential positive effect for 2009 type A H1N1 influenza on fever resolution, relief of flu-like symptoms, and global effectiveness rate. However, due to the lack of placebo controlled trial and lack of repeated test, we could not make confirmative conclusions on the therapeutic effect of Chinese herbs for H1N1 influenza. In addition, we found out that few Chinese herbs recommended in the ‘Guidelines for Management of Pandemic (H1N1) 2009 Influenza’ issued by the Ministry of Health of China were supported in this review, which revealed the lack of evidence for clinical use and policy making in China.

The following reasons might contribute to the inconclusive results of Chinese herbs. Firstly, all the included trials were of poor methodology quality, which were in accordance with previous studies [53], [54]. Only six RCTs stated randomization procedure, however, most of them provided insufficient information to judge whether randomization was conducted properly. For the rest fourteen trials, they just mentioned that ‘the patients were randomized into two groups’ with no further information. Allocation concealment was only mentioned in one RCT. Therefore, we could not exclude the possibility that some of these claimed RCTs are not real RCTs. This possibility came to forefront in the trial of Han 2011 and Li 2010 [11], [13]; these trials only has one author, which is impossible for a RCT to be done properly in terms of randomization procedure and the allocation concealment. In addition, no trials claimed double-blind. We understood that it was difficult to perform double-blinding because of certain features associated with Chinese herbs, for example, aroma and appearance, but blinding to the outcome assessors and data analyzer could be feasible. Unfortunately, none of the RCTs mentioned blinding to the outcome assessors or data analyzer. All the trials did not report pre-sample size estimation except one [24], and for majority trials, the sample size was small. Therefore we are not sure if they could provide enough power to detect the difference between groups. It is well known that methodologically poorly designed trials show larger differences between experimental and control groups than those conducted rigorously [55]–[57] and as such the small improvements in outcomes should be regarded with caution.

Secondly, there was lack of knowledge for placebo control in the included trials. Two trials [16], [27] claimed that they used placebo control. However, in these trials the placebo is for oseltamivir, not for Chinese herbs. It is not an appropriate control for the estimation of the effect of Chinese herbs. Because of the lack of placebo controls, the interpretation of the positive findings of treatment with Chinese herbs should be made with caution. In addition, the potential positive placebo effect of an injection should also be highlighted. In the review a total of three Chinese herbal injections were used, that is, Tanreqing Injection [11], [13], Xiyanping Injection [22], and Reduning Injection [26]; and all demonstrated positive results in terms of defervescence and global symptoms improvement. However, no adequate placebo control was used to offset the effect of injection alone. It is known that an injection alone has a strong potential placebo effect, therefore the overall effect of Chinese herbal injection could not rule out the effect that the injection itself produced. These positive effects should also be interpreted conservatively.

Thirdly, in most of the trials, the patients were not treated according to syndrome differentiation. In the practice of TCM, herbal preparations should match the type of syndrome differentiation, that is, ‘bianzheng’, a specific diagnosis in TCM. This approach is also known as treatment based on individualized (tailored) syndrome pattern, and is thought to be the advantages of TCM. However, in this review, only seven trials provide information on patients' syndrome differentiation. Chinese medicine practitioners believed that treating patients without syndrome differentiation will impair the advantages of Chinese herbs, and this might be another reason for the unsatisfactory therapeutic effect of Chinese herbs for H1N1 influenza in the review. Emphasis should be paid to encourage authors to explain each ‘Bianzheng’ by using common medical terms in the future trials, therefore making it understandable by physicians and consumers.

Finally, there existed great heterogeneity in the Chinese herbs investigated in the review. A total of 27 different Chinese herbs were investigated in the 26 trials. As a result, it is impossible to conduct meaningful meta-analysis for a specific Chinese herb, or difficult to undertake subgroup analyses to explore specific factors that may have an impact on the effects of the treatment regimen. What is more interesting is that although diversified Chinese herbs were investigated, most of them were not the authoritatively recommended Chinese herbs for the H1N1 influenza. In October 2009, the Ministry of Health of China issued ‘Guidelines for Management of Pandemic (H1N1) 2009 Influenza A’ and recommended series of Chinese herbs for the treatment of H1N1 influenza A [5]. These Chinese herbs included herbal products extracted from natural herbs, Chinese patent medicines (including herbal injection), and principles for prescribing individually herbal decoction. However, only three Chinese patent medicines (Lianhuaqingwen Capsule [12], [15], [17], [18], Xiyanping Injection [22], Tanreqing Injection [11], [13], [19]) and two prescript Chinese herbal decoction (unnamed Self-prescribed Chinese herbal decoction [9], and Qingfei Jiedu Decoction [10]) in the guideline had been investigated by RCTs, and only two of them (Tanreqing Injection [11], [13], [19] and Qingfei Jiedu Decoction [10]) demonstrated positive results. World Health Organization (WHO) calls for evidence-based practice of TCM, that is, any medical decision-making of TCM should be based on clinical research evidence. In the era of evidence-based medicine (EBM), TCM is facing a big challenge because of the lack of a rigorous research evidence base. Our review revealed the lack of evidence for clinical use and policy making of Chinese herbs for H1N1 influenza in China. There is still a long way to go for evidence-based practice of TCM.

In addition, our review found inadequate reporting on adverse events in the included trials. Eleven trials did not mention whether they had monitored adverse effects at all. Conclusions about the safety of herbal medicines cannot be drawn from this review due to the limited, inadequate recording and reporting of adverse events. Even for the trials that reported adverse events, their report was very brief, providing limited information. In China, there is a general perception that it is safe to use herbal medicines for various conditions. However, with the increasing reports of liver toxicity and other adverse events associated with Chinese herbal medicines [58]–[60], more emphasis should be placed on the monitoring and reporting of adverse events to justify the safety of Chinese herbs in clinical trials in the future.

The funnel plot analysis showed asymmetry which suggests the possibility of publication bias of Chinese herbs for H1N1 influenza. Almost all the trials claimed positive effect of Chinese herbs though some of them turned out to be negative when analyzed by standard statistical techniques using risk ratios or mean differences. We undertook extensive searches for unpublished material, but found no unpublished ‘negative’ studies as in previous study [61]. We thought this might be attributed to the lack of awareness to register clinical trials in China, the rejection of journal editors to negative trials, and the inaccessibility to unpublished data. We hope that with increasing awareness of prospective registration of clinical trials, publication of clinical trial protocol and reporting of negative clinical trials, the picture may change in the future.

The mechanism of Chinese herbs in the treatment of influenza is complex. In traditional Chinese medicine (TCM), H1N1 influenza belongs to the scope of ‘cold’. In TCM, cold is differentiated into two types: Wind-cold Syndrome and Wind-heat Syndrome. The principles behind treating these two types were different. Generally speaking, the principles behind treating wind-cold syndrome are to: relieve external symptoms with drugs which are pungent in flavour and warm in property; ventilate the lungs and expel the pathogenic cold. The principles behind treating wind-heat syndrome are to: relieve external symptoms with drugs which are pungent in flavour and cool in property and promote the dispersing function of the lungs and clear up pathogenic wind heat [62], [63]. In addition, previous studies showed that administration of some Chinese herbs may have beneficial immunomodulatory effects for rapid recovery of viral infections [64], [65]. However, in this review, it seems that compared with oseltamivir, Chinese herbs might have better potential effects on fever solution than viral shedding, which suggested that most of Chinese herbs might not act as an antiviral.

Last but not least, there is a lack of information about quality control for the development of the herbal preparations or for the manufacture of the herbal products, which is a quite common problem in Chinese clinical trials. Future trials should provide information about standardization including compositions, quality control, detailed regimen, and duration of treatment.

In summary, the reported beneficial effect from Chinese herbs for H1N1 influenza can not be taken as confirmative conclusion. To ensure evidence-based clinical practice, further rigorous placebo-controlled, randomized trials are warranted. The following methodological issues should be addressed: (i) methods used to generate allocation sequence and allocation concealment; (ii) double blinding with the use of adequate placebo; (iii) clear descriptions of withdrawal/dropout during the trial and use of intention-to-treat analysis; and (iv) reporting trials according to the CONSORT Statement (www.consort_statement.org) [57]. In the literature searching, we identified several registered trials of Chinese herbs for H1N1 influenza. We hope with the publication of these ongoing trials in the future, new high-qualified evidence will arise to provide clinical evidence for the use of Chinese herbs for the H1N1 influenza.

## Supporting Information

Figure S1 **Forest plot of comparison of CHM versus oseltamivir for the outcome of duration of fever.**
(TIF)

Figure S2 **Funnel plot of comparison of CHM versus oseltamivir for the outcome of duration of fever.**
(TIF)

Table S1 **Characteristics of included RCTs.**
(DOC)

Table S2 **Effect estimates of Chinese herbs for treatment of H1N1 influenza in included trials.**
(DOC)

Table S3 **Excluded studies with reasons.**
(DOC)

Table S4 **Compositions of Chinese herbs in the included trials.**
(DOC)

Checklist S1 **PRISMA 2009 checklist.**
(DOC)
