# Supplementary material for: Chinese Herbal Medicines for the Treatment of Type A H1N1 Influenza: A Systematic Review of Randomized Controlled Trials
Source: PLoS One. 2011 Dec 2;6(12):e28093. doi: 10.1371/journal.pone.0028093 (PMC3229517; doi:10.1371/journal.pone.0028093)
Supplement: Table S1 — Characteristics of included RCTs. (DOC) [file pone.0028093.s003.doc]

## Table S1. Characteristics of included RCTs

| **Study ID** | **No. (M/F)** | **Age (yrs)** | **Pattern of syndromes** | **Comparisons** | **Outcome measures** | **Adverse events** |
| --- | --- | --- | --- | --- | --- | --- |
| Chen 2010^7^ | T:18/13; C:9/13 | T:19.87±9.2; C:20.68±6.97 | Pattern of wind-warm attacked on surface of body; damp-worm pattern of syndrome; pattern of heat toxin in qi level | Mixed Chinese herbs (Modified Yinqiao Powder, Huopu Xialing Decoction, Modified Puji Xiaodu Decoction, Sangju Decoction) vs. oseltamivir for treatment of 5 days. | Duration of fever; duration of flu-like symptoms; hospitalization duration | One case of diarrhea identified in Chinese herbs group; one case of eruption, two cases of nausea and vomit in control group. |
| Chen 2010a^8^ | 56/39（for all participants） | 18±5.7（for all participants） | Pattern of wind-warm invading defense level | FanGan Granule vs. symptomatic treatment for treatment of 3-5 days. | Duration of fever; duration of flu-like symptoms; global improvement rate | One case of diarrhea and one case of arrhythmia identified in Chinese herbs group; one case of pneumonia and one case of chest pain in control group. |
| Dou 2010^9^ | 98/89（for all participants） | 20.61±13.87（for all participants） | Not reported | Self-prescribed Chinese herbs vs. oseltamivir for treatment of 5 days. | Duration of fever; duration of flu-like symptoms; duration of viral shedding; global improvement rate | Not reported. |
| Jin 2010^10^ | T:19/15; C:not reported | T:40±8; C:not reported | Pattern of heat-toxin attack on lung | Qingfei Jiedu Decoction vs. oseltamivir (treatment duration not reported) | Global improvement rate | One case of mild upper abdominal discomfort and two cases of nausea and vomit in control group. |
| Han 2011^11^ | T:22 **(**M/F not reported); C:21 **(**M/F not reported) | 35.5 （for all participants） | Not reported | Tanreqing Injection plus oseltamivir vs. oseltamivir for treatment of 5 days. | Global improvement rate | Not reported. |
| Li 2009^12^ | T:11/14; C:9/16 | T:19; C:18 | Not reported | Lianhuaqingwen Capsule vs. oseltamivir for treatment of 5 days. | Duration of fever; duration of viral shedding | No adverse event was identified. |
| Li 2010^13^ | T:28/27; C:32/23 | T:31.35; C:30.77 | Not reported | Tanreqing Injection plus oseltamivir vs. oseltamivir for treatment of 7-14 days. | Duration of fever; global improvement rate | Not reported. |
| Lin 2011^14^ | 61/19（for all participants） | 26.3±4.3（for all participants） | Pattern of wind-warm invading defense level; Pattern of heat-toxin attack on lung | Xiaochaihu Decoction vs. oseltamivir for treatment of 5 days. | Duration of flu-like symptoms; duration of viral shedding; hospitalization duration | No adverse event was identified. |
| Liu 2010^15^ | T:34/30; C:35/25 | T:19.8±3.7; C:19.6±1.4 | Not reported | Lianhuaqingwen Capsule vs. oseltamivir for treatment of 5 days. | Duration of fever; duration of viral shedding; hospitalization duration | No adverse event was identified. |
| Liu 2011^16^ | T:18/13; C1:9/13; C2:13/9 | T:19.87±9.20; C1:20.68±1.48; C2:20.09±5.64 | Not reported | Chinese herb plus placebo of oseltamivir (T) vs. oseltamivir (C1) vs. placebo of oseltamivir (C2) for treatment of 5 days. | Duration of fever; duration of flu-like symptoms; hospitalization duration | One case of mild diarrhea in Chinese herbs group; two cases of eruption and two cases of vomit in control group. |
| Ma 2010^17^ | T1:45/28; T2:37/36; T3:42/18; C:47/27 | T1:22.8±6.3; T2:23.9±7.9; T3:23.6±8.8; C:23.2±8.7 | Not reported | Self-prescribed Chinese herbs (T1) vs. Self-prescribed Chinese herbs plus oseltamivir (T2) vs. Lianhuaqingwen Capsule (T3) vs. oseltamivir (C) for treatment of 5-7 days. | Duration of fever; duration of viral shedding; hospitalization duration; global improvement rate; | Four cases of mild nausea and mild upper abdominal discomfort in oseltamivir group; two cases of mild nausea and mild upper abdominal discomfort in Chinese herbs plus oseltamivir group. |
| Ou 2010^18^ | T:59/57; C:16/13 | T:19.23±10.44; C:19.69±9.91 | Pattern of heat-toxin attack on lung | Lianhuaqingwen Capsule vs. oseltamivir for treatment of 5 days. | Global improvement rate; | Lung infection identified in three cases in Lianhuaqingwen group and in one case in oseltamivir group. |
| Qian 2011^19^ | T:11/14; C:16/13 | T:40.91±19.81; C:41.22±15.62 | Not reported | Tanreqing Injection plus oseltamivir vs. oseltamivir (treatment duration not reported) | Duration of fever; duration of viral shedding; hospitalization duration | Not reported. |
| Qu 2010^20^ | T:16/14; C:14/16 | T:21.33±7.36; C:21.16±8.8 | Not reported | Bingyanqing formula Ten vs. oseltamivir (treatment duration not reported) | Duration of fever | Not reported. |
| Tan 2010^21^ | T1:29; T2:42; C1:43; C2:15 | T1:21±11.32; T2:21±11.06; C1:22±10.95; C2:18.2±4.69 | Not reported | Mixed Chinese herbs (Modified Chaige Jieji Decoction, Yinhuang Granules, Shuanghuagnlian Oral Liquid) (T1) vs. combination of mixed Chinese herbs and oseltamivir (T2) vs. oseltamivir (C1) vs. symptomatic treatment (C2) for treatment of 3-5 days. | Duration of fever; duration of viral shedding | Not reported. |
| Tang 2010^22^ | T:19/11; C:16/14 | T:20.5; C:21.2 | Not reported | Xiyanping Injection plus oseltamivir vs. oseltamivir for treatment of 4 days. | Global improvement rate | Two cases of wheal identified, but do not reported in which group. |
| Tian 2011^23^ | T:16/24; C:12/8 | T:22.9±4.4; C:22.9±6.0 | Not reported | Qingkailing Oral Liquid vs. oseltamivir for treatment of 7 days. | Duration of fever; duration of flu-like symptoms | Not reported. |
| Wang 2011^24^ | T1:65/38; T2:52/50; C1:58/44; C2:58/45 | T1:19.6±7.1; T2:19.2±6.5; C1:19.0±6.2; C2:18.7±5.3 | Not reported | Maxingshigan–yinqiaosan decoction (T1) vs. oseltamivir plus maxingshigan–yinqiaosan (T2) vs. oseltamivir (C1) vs. no intervention (C2) for treatment of 5 days. | Duration of fever; Proportion of patients became afebrile (body temperature ≤37℃ for ≥24 hours) | Two patients in the maxingshigan–yinqiaosan group had nausea and vomiting. |
| Weng 2010^25^ | T:95/55; C:90/60 | T:10.5; C:11.2 | Not reported | Qingjie Huashi Decoction vs. oseltamivir for treatment of 5 days. | Global improvement rate | Six cases of mild nausea, three cases of diarrhea in control group. |
| Ye 2010^26^ | T:58/42; C:48/42 | T:2-16; C:4-15 | Not reported | Reduning Injection plus ribavirin and oseltamivir vs. ribavirin and oseltamivir for treatment of 5-7 days. | Global improvement rate | Not reported. |
| Zeng 2011^27^ | T1:18/13; T2:13/15; C1:10/19; C2:15/11 | T1:18.52±7.70; T2:17.89±4.06; C1:19.48±6.46; C2:19.62±5.58 | Not reported | Maxingshigan Decoction plus placebo of oseltamivir (T1) vs. Maxingshigan Decoction plus oseltamivir (T2) vs. oseltamivir (C1) vs. placebo of oseltamivir (C2) for the treatment of 5 days. | Duration of fever; duration of flu-like symptoms; global improvement rate; hospitalization duration | One case of eruption and two cases of nausea and vomit in oseltamivir group. |
| Zhang 2011^28^ | T:17/13; C:16/14 | T:22.77± 3.87; C:23.37± 3.98 | Pattern of wind-heat invasion of lung | Self-prescribed Chinese herbs vs. oseltamivir for treatment of 5 days. | Duration of fever; duration of viral shedding; global improvement rate | Not reported. |
| Zhao 2010^29^ | T:18/12; C:16/14 | T:18.20± 2.63; C:16.27± 3.23 | Not reported | Qingwen Tuire Decoction plus oseltamivir vs. oseltamivir for treatment of 5 days. | Duration of fever; duration of flu-like symptoms | Not reported. |
| Zhao 2011^30^ | T:27/4; C:14/2 | T:18.97± 2.88; C:20.06± 2.86 | Not reported | Self-prescribed Chinese herbs vs. oseltamivir. The treatment duration was not reported. | Duration of viral shedding | WBC decreased in 5 cases in the control group. Desudation and diarrhea in intervention group without detailed information. |
| Zheng 2010^31^ | T1:7/7; T2:9/6; C:12/7 | T1:19.79±15.29; T2:23.53±8.48; C:22.47±9.40 | Not reported | Yinqiao Powder, Sanao Decoction (T1) vs. combination of Yinqiao Powder, Sanao Decoction and oseltamivir (T2) vs. oseltamivir (C) for treatment of 6 days. | Duration of fever; duration of flu-like symptoms; duration of viral shedding | No adverse event was identified. |
| Zhu 2010^32^ | T:25/13; C:22/10 | T:35.12; C:34.23 | Pattern of external wind cold invasion | Gegen Granules plus oseltamivir vs. Oseltamivir for treatment of 5 days. | Duration of fever; | Not reported. |

**Abbreviations:** T, Intervention group; C, control group.
