# Supplementary material for: Chinese Herbal Medicines for the Treatment of Type A H1N1 Influenza: A Systematic Review of Randomized Controlled Trials
Source: PLoS One. 2011 Dec 2;6(12):e28093. doi: 10.1371/journal.pone.0028093 (PMC3229517; doi:10.1371/journal.pone.0028093)
Supplement: Table S2 — Effect estimates of Chinese herbs for treatment of H1N1 influenza in included trials. (DOC) [file pone.0028093.s004.doc]

**Table S2.** Effect estimates of Chinese herbs for treatment of H1N1 influenza in included trials

## Abbreviations: CI, confidence interval; *, the effect estimate favors experimental group; #, the effect estimate favors control group; ∆, result from Meta-analysis.

| **Comparisons** | **Effect estimate (95% CI)** | **Study ID** |
| --- | --- | --- |
| **Duration of fever (hours)** | | |
| Mixed Chinese herbs vs. oseltamivir | MD 3.44 (2.31, 4.57) # | Chen 2010^7^ |
| Self-prescribed Chinese herbs vs. oseltamivir | MD 0.15 (-0.65, 0.95) | Dou 2010^9^ |
| Self-prescribed Chinese herbs plus placebo of oseltamivir vs. placebo of oseltamivir | MD -5.85 (-15.90, 4.20) | Liu 2011^16^ |
| Self-prescribed Chinese herbs plus placebo of oseltamivir vs. oseltamivir | MD -0.73 (-6.44, 4.98) | Liu 2011^16^ |
| Lianhuaqingwen Capsule vs. oseltamivir | MD -0.04 (-0.28, 0.20) ^∆^ | Li 2009^12^, Ma 2010^17^ |
| Self-prescribed Chinese herbs vs. oseltamivir | MD -0.27 (-0.48, -0.06) * | Ma 2010^17^ |
| Self-prescribed Chinese herbs plus oseltamivir vs. oseltamivir | MD -0.31 (-0.52, -0.10) * | Ma 2010^17^ |
| Bingyanqing formula Ten vs. oseltamivir | MD -8.95 (-15.83, -2.07) * | Qu 2010^20^ |
| Mixed Chinese herbs vs. oseltamivir | MD 3.44 (-4.03, 10.91) | Tan 2010^21^ |
| Mixed Chinese herbs plus oseltamivir vs. oseltamivir | MD -3.97 (-10.47, 2.53) | Tan 2010^21^ |
| Mixed Chinese herbs vs. symptomatic treatment | MD -2.07 (-11.21, 7.07) | Tan 2010^21^ |
| Maxingshigan decoction plus oseltamivir vs. oseltamivir | MD 0.45 (-0.85, 1.75) | Zeng 2007^27^ |
| Maxingshigan decoction plus oseltamivir vs. placebo of oseltamivir | MD -8.87 (-9.93, -7.81) * | Zeng 2007^27^ |
| Maxingshigan decoction plus placebo of oseltamivir vs. oseltamivir | MD 4.24 (3.13, 5.35) # | Zeng 2007^27^ |
| Maxingshigan decoction plus placebo of oseltamivir vs. placebo of oseltamivir | MD -5.08 (-5.89,-4.27) * | Zeng 2007^27^ |
| Self-prescribed Chinese herbs vs. oseltamivir | MD -11.96 (-12.98, -10.94) * | Zhang 2011^28^ |
| Qingwen Tuire Decoction plus oseltamivir vs. oseltamivir | MD -5.40 (-11.29, 0.49) | Zhao 2010^29^ |
| Gegen Granule plus oseltamivir vs. oseltamivir | MD -6.44 (-10.29, -2.59) * | Zhu 2010^32^ |
| **Duration of fever (days)** | | |
| Fanggan Granule vs. symptomatic treatment | MD -1.01 (-1.52, -0.50) * | Chen 2010a^8^ |
| Lianhuaqingwen Capsule vs. oseltamivir | MD 0.10 (-0.22, 0.42) | Liu 2010^15^ |
| Tanreqing Injection plus oseltamivir vs. oseltamivir | MD -1.18 (-1.46, -0.90) *^∆^ | Li 2010^13^, Qian 2011^19^ |
| Qingkailing Oral Liquid vs. oseltamivir | MD 1.88 (1.16, 2.60) **#** | Tian 2011^23^ |
| Mixed Chinese herbs vs. oseltamivir | MD 1.11 (0.18, 2.04) # | Zheng 2010^31^ |
| Mixed Chinese herbs plus oseltamivir vs. oseltamivir | MD 0.64 (0.07, 1.21) # | Zheng 2010^31^ |
| **Proportion of patients became afebrile (body temperature ≤37℃ for ≥24 hours)** |  |  |
| Maxingshigan-yinqiaosan vs. no intervention | RR 1.03 (0.92, 1.16) | Wang 2011^24^ |
| Maxingshigan-yinqiaosan plus oseltamivir vs. no intervention | RR 1.16 (1.06, 1.27) * | Wang 2011^24^ |
| Maxingshigan-yinqiaosan vs. no oseltamivir | RR 0.92 (0.84, 1.01) | Wang 2011^24^ |
| Maxingshigan-yinqiaosan plus oseltamivir vs. oseltamivir | RR 1.04 (0.99, 1.10) | Wang 2011^24^ |
| **Duration of flu-like symptom (hours)** | | |
| Mixed Chinese herbs vs. oseltamivir | MD -2.64 (-16.52, 11.24) | Chen 2010^7^ |
| Self-prescribed Chinese herbs plus placebo vs. placebo | MD -16.26 (-42.32, 9.80) | Liu 2011^16^ |
| Self-prescribed Chinese herbs plus placebo vs. oseltamivir | MD -7.54 (-23.08, 8.00) | Liu 2011^16^ |
| Maxingshigan decoction plus oseltamivir vs. oseltamivir | MD 7.00 (4.12, 9.88) # | Zeng 2007^27^ |
| Maxingshigan decoction plus oseltamivir vs. placebo of oseltamivir | MD 11.32 (8.44, 14.20) # | Zeng 2007^27^ |
| Maxingshigan decoction plus placebo of oseltamivir vs. oseltamivir | MD 0.10 (-2.29, 2.49) | Zeng 2007^27^ |
| Maxingshigan decoction plus placebo of oseltamivir vs. placebo | MD 4.42 (2.03, 6.81) # | Zeng 2007^27^ |
| Qingwen Tuire Decoction plus oseltamivir vs. oseltamivir | MD -13.33 (-24.28, -2.38) * | Zhao 2010^29^ |
| **Duration of flu-like symptom (days)** | | |
| Fanggan Granule vs. symptomatic treatment | MD -1.24 (-1.71, -0.77) * | Chen 2010a^8^ |
| Self-prescribed Chinese herbs vs. oseltamivir | MD 0.56 (-0.38, 1.50) | Dou 2010^9^ |
| Xiaochaihu Decoction vs. oseltamivir | MD 0.27 (-0.43, 0.97) | Lin 2011^14^ |
| Qingkailing Oral Liquid vs. oseltamivir | MD 0.67 (-0.15, 1.49) | Tian 2011^23^ |
| Mixed Chinese herbs vs. oseltamivir | MD 0.68 (-0.24, 1.60) | Zheng 2010^31^ |
| Mixed Chinese herbs plus oseltamivir vs. oseltamivir | MD 0.44 (-0.69, 1.57) | Zheng 2010^31^ |
| **Global improvement rate** | | |
| Fanggan Granule vs. symptomatic treatment | RR 1.05 (0.90, 1.23) | Chen 2010a^8^ |
| Qingfei Jiedu Decoction vs. oseltamivir | RR 1.43 (1.07, 1.91) * | Jin 2010^10^ |
| Tanreqing Injection plus oseltamivir vs. oseltamivir | RR 1.38 (1.14, 1.66) *^∆^ | Han 2011^11^, Li 2010^13^ |
| Lianhuaqingwen Capsule vs. oseltamivir | RR 1.00 (0.91, 1.10) ^∆^ | Ma 2010^17^, Ou 2010^18^ |
| Self-prescribed Chinese herbs vs. oseltamivir | RR 1.00 (0.92, 1.08) | Ma 2010^17^ |
| Self-prescribed Chinese herbs plus oseltamivir vs. oseltamivir | RR 0.97 (0.89, 1.06) | Ma 2010^17^ |
| Xiyanping Injection plus oseltamivir vs. oseltamivir | RR 1.40 (1.07, 1.83) * | Tang 2010^22^ |
| Qingjie Huashi Decoction vs. oseltamivir | RR 1.24 (1.07, 1.44) * | Weng 2010^25^ |
| Reduning Injection plus ribavirin and oseltamivir vs. ribavirin and oseltamivir | RR 1.09 (1.01, 1.18) * | Ye 2010^26^ |
| Maxingshigan decoction plus oseltamivir vs. oseltamivir | RR 0.96 (0.88, 1.06) | Zeng 2007^27^ |
| Maxingshigan decoction plus oseltamivir vs. placebo of oseltamivir | RR 1.04 (0.94, 1.15) | Zeng 2007^27^ |
| Maxingshigan decoction plus placebo of oseltamivir vs. oseltamivir | RR 1.00 (0.94, 1.07) | Zeng 2007^27^ |
| Maxingshigan decoction plus placebo of oseltamivir vs. placebo | RR 1.04 (0.94, 1.15) | Zeng 2007^27^ |
| Self-prescribed Chinese herbs vs. oseltamivir | RR 1.20 (0.88, 1.64) | Zhang 2011^28^ |
| **Hospitalization duration (days)** | | |
| Mixed Chinese herbs vs. oseltamivir | MD -0.04 (-0.71, 0.63) | Chen 2010^7^ |
| Xiaochaihu Decoction vs. oseltamivir | MD 0.38 (-0.49, 1.25) | Lin 2011^14^ |
| Self-prescribed Chinese herbs plus placebo vs. placebo | MD -0.02 (-0.90, 0.86) | Liu 2011^16^ |
| Self-prescribed Chinese herbs plus placebo vs. oseltamivir | MD -0.04 (-0.59, 0.51) | Liu 2011^16^ |
| Self-prescribed Chinese herbs vs. oseltamivir | MD -0.30 (-0.50, -0.10) * | Ma 2010^17^ |
| Self-prescribed Chinese herbs plus oseltamivir vs. oseltamivir | MD -0.10 (-0.23, 0.03) | Ma 2010^17^ |
| Lianhuaqingwen Capsule vs. oseltamivir | MD -0.10 (-0.25, 0.05) | Ma 2010^17^ |
| Tanreqing Injection plus oseltamivir vs. oseltamivir | MD -1.01 (-2.00, -0.02) * | Qian 2011^19^ |
| Maxingshigan decoction plus oseltamivir vs. oseltamivir | MD -0.10 (-0.73, 0.53) | Zeng 2007^27^ |
| Maxingshigan decoction plus oseltamivir vs. placebo of oseltamivir | MD 0.04 (-0.62, 0.70) | Zeng 2007^27^ |
| Maxingshigan decoction plus placebo of oseltamivir vs. oseltamivir | MD 0.13 (-0.51, 0.77) | Zeng 2007^27^ |
| Maxingshigan decoction plus placebo of oseltamivir vs. placebo | MD 0.27 (-0.40, 0.94) | Zeng 2007^27^ |
| **Duration of viral shedding (days)** | | |
| Self-prescribed Chinese herbs vs. oseltamivir | MD 0.49 (-0.39, 1.37) | Dou 2010^9^ |
| Lianhuaqingwen Capsule vs. oseltamivir | MD -0.12 (-0.34, 0.10) ^∆^ | Li 2009^12^, Liu 2010^15^, Ma 2010^17^ |
| Xiaochaihu Decoction vs. oseltamivir | MD 0.54 (-0.36, 1.44) | Lin 2011^14^ |
| Self-prescribed Chinese herbs vs. oseltamivir | MD -0.10 (-0.24, 0.04) | Ma 2010^17^ |
| Self-prescribed Chinese herbs plus oseltamivir vs. oseltamivir | MD -0.10 (-0.26, 0.06) | Ma 2010^17^ |
| Tanreqing Injection plus oseltamivir vs. oseltamivir | MD -0.54 (-1.14, 0.06) | Qian 2011^19^ |
| Mixed Chinese herbs vs. symptomatic treatment | MD -0.36 (-1.30, 0.58) | Tan 2010^21^ |
| Mixed Chinese herbs vs. oseltamivir | MD 0.34 (-0.26, 0.94) | Tan 2010^21^ |
| Mixed Chinese herbs plus oseltamivir vs. oseltamivir | MD 0.32 (-0.25, 0.89) | Tan 2010^21^ |
| Mixed Chinese herbs plus oseltamivir vs. oseltamivir | MD 0.92 (-0.09, 1.93) | Zheng 2010^31^ |
| Mixed Chinese herbs vs. oseltamivir | MD 1.49 (-0.06, 3.04) | Zheng 2010^31^ |
| Self-prescribed Chinese herbs vs. oseltamivir | MD -1.83 (-2.20, -1.46) * | Zhang 2011^28^ |
| Self-prescribed Chinese herbs vs. oseltamivir | MD 0.94 (-0.01, 1.98) | Zhao 2011^30^ |
