# Supplementary material for: Chinese Herbal Medicines for the Treatment of Type A H1N1 Influenza: A Systematic Review of Randomized Controlled Trials
Source: PLoS One. 2011 Dec 2;6(12):e28093. doi: 10.1371/journal.pone.0028093 (PMC3229517; doi:10.1371/journal.pone.0028093)
Supplement: Table S3 — Excluded studies with reasons. (DOC) [file pone.0028093.s005.doc]

## Table S3. Excluded studies with reasons

| **Study** | **Reason for exclusion** |
| --- | --- |
| Chai 2010^33^ | Retrospective study that did not match the included criteria of this review |
| Hu 2011^34^ | Patients was complicated with pneumonia |
| Huang 2010^35^ | Patients in the trials was suspected H1N1 influenza patients |
| Kou 2011^36^ | Patients was complicated with pneumonia |
| Li 2010^37^ | Randomized controlled trial but data were not available for analysis due to inadequate reporting |
| Li 2010^38^ | Retrospective study that did not match the included criteria of this review |
| Liu 2010^39^ | Randomized controlled trial that another Chinese herbs was taken as the control intervention |
| Liu 2010^40^ | Randomized controlled trial but data were not available for analysis due to inadequate reporting |
| Qin 2010^41^ | Randomized controlled trial that another Chinese herbs was taken as the control intervention |
| Qiu 2010^42^ | Prophylactic randomized controlled trial that investigated Chinese herbs in the prevention of A/H1N1 influenza in pregnant women |
| Ru 2010^43^ | Randomized controlled trial that another Chinese herbs was taken as the control intervention |
| Song 2010^44^ | Randomized controlled trial that was a replicated publication of Chen 2010a^8^ |
| Wei 2010^45^ | Randomized controlled trial but data were not available for analysis due to inadequate reporting |
| Wen 2010^46^ | Randomized controlled trial that another Chinese herbs was taken as the control intervention |
| Xia 2010^47^ | Randomized controlled trial that another Chinese herbs was taken as the control intervention |
| Xie 2010^48^ | Randomized controlled trial that another Chinese herbs was taken as the control intervention |
| Xie 2010^49^ | Quasi-randomized controlled trial that did not match the included criteria of this review |
| Yang 2010^50^ | Randomized controlled trial that another Chinese herbs was taken as the control intervention |
| Zhang 2010^51^ | Randomized controlled trial that another Chinese herbs was taken as the control intervention |
