# Supplementary material for: Chinese Herbal Medicines for the Treatment of Type A H1N1 Influenza: A Systematic Review of Randomized Controlled Trials
Source: PLoS One. 2011 Dec 2;6(12):e28093. doi: 10.1371/journal.pone.0028093 (PMC3229517; doi:10.1371/journal.pone.0028093)
Supplement: Table S4 — Compositions of Chinese herbs in the included trials. (DOC) [file pone.0028093.s006.doc]

**Table S4. Compositions of Chinese herbs in the included trials**

| **study ID** | **Name of Chinese herbs** | **Compositions** |
| --- | --- | --- |
| Chen 2010^7^ | Modified Yinqiao Powder; Huopu Xialing Decoction; Modified Puji Xiaodu Decoction; Sangju Decoction | Modified Yinqiao Powder (Flos Lonicerae 30g, Fructus Forsythiae 30g, Radix Platycodonis 15g, Fructus Arctii 15g, Herba Menthae 15g, Herba Schizonepetae 15g, Rhizoma Phragmitis 30g, Folium Eriobotryae 15g, Lasiosphaera seu Calvatia 15g, Radix Scrophulariae 30g, Raw Radix Glycyrrhizae 10g); Huopu Xialing Decoction (Herba Pogostemonis 15g, Cortex Magnoliae Officinalis 15g, Rhizoma Pinelliae Preparatum 15g, Poria 15g, Semen Coicis 30g, Semen Lablab 30g, Fructus Amomi 15g, Rhizoma Atractylodis 30g, Pericarpium Citri Reticulatae 15g, Rhizoma Coptidis 15g, Burnt Fructus Crataegi 30g, Radix Glycyrrhizae 10g); Modified Puji Xiaodu Decoction (Flos Lonicerae 30g, Fructus Forsythiae 30g, Radix Platycodonis 15g, Radix Isatidis 30g, Radix Scrophulariae 30g, Radix Bupleuri 15g, Herba Menthae 15g, Fructus Arctii 15g, Lasiosphaera seu Calvatia 15g, Radix Scutellariae 15g, Radix Glycyrrhizae 10g, Raw Rhizoma Cimicifugae 12g); Sangju Decoction (Folium Mori 15g, Hang Flos Chrysanthemi 15g, Radix Platycodonis 15g, Fructus Forsythiae 15g, Rhizoma Phragmitis 30g, Radix Glehniae 30g, Herba Menthae 15g, Radix Ophiopogonis 15g, Radix Glycyrrhizae 12g, Semen Armeniacae Amarum 12g) |
| Chen 2010a^8^ | FanGan Granule | Herba Schizonepetae 10g, Radix Peucedani 10g, Radix Isatidis 10g, Folium Isatidis 10g, etc. |
| Dou 2010^9^ | Self-prescribed Chinese herbs | Flos Lonicerae 15g, Fructus Forsythiae 15g, Folium Mori 10g, Hang Flos Chrysanthemi 10g, Radix Platycodonis 10g, Fructus Arctii 15g, Bamboo leaves 6g, Rhizoma Phragmitis 30g, Herba Menthae 3g, Raw Radix Glycyrrhizae 3g. |
| Jin 2010^10^ | Qingfei Jiedu Decoction | Baked Herba Ephedrae 3g, Semen Armeniacae Amarum 10g, Gypsum Fibrosum 20g, Raw Radix Glycyrrhizae 10g, Bulbus Fritillariae Thunbergii 10g, Radix Scutellariae 15g, Radix Bupleuri 15g, Radix Platycodonis 15g, Rhizoma Anemarrhenae 10g, Fructus Trichosanthis 15g. |
| Han 2011^11^ | Tanreqing Injection | Radix Scutellariae, fel ursi powder, cornu caprae hircus, Flos Lonicerae, Fructus Forsythiae. |
| Li 2009^12^ | Lianhuaqingwen Capsule | Fructus Forsythiae, Flos Lonicerae, Baked Herba Ephedrae, Stir-baked Semen Armeniacae Amarum, Gypsum Fibrosum, Radix Isatidis, Rhizoma Dryopteris Crassirhizomae, Herba Houttuyniae, Herba Pogostemonis, Radix et Rhizoma Rhei, Herba Rhodiolae, menthol, Radix Glycyrrhizae. |
| Li 2010^13^ | Tanreqing Injection | The same as Han 2011^11^. |
| Lin 2011^14^ | Xiaochaihu Decoction | Radix Bupleuri 20g, Radix Scutellariae 10g, Rhizoma Pinelliae 10g, Radix Pseudostellariae 10g, Rhizoma Zingiberis Recens 10g, Fructus Jujubae 10g, prepared Radix Glycyrrhizae 6g. Raw Gypsum Fibrosum, Flos Lonicerae, Fructus Forsythiae, and Folium Isatidis were added additionally. |
| Liu 2010^15^ | Lianhuaqingwen Capsule | The same as Li 2009^12^. |
| Liu 2011^16^ | Self-prescribed Chinese herbs | The compositions of Chinese herbs were not provided. |
| Ma 2010^17^ | Self-prescribed Chinese herbs; Lianhuaqingwen Capsule | Self-prescribed Chinese herbs (Flos Lonicerae 15g, Fructus Forsythiae 15g, Flos Chrysanthemi 10g, Fructus Arctii 10g). Lianhuaqingwen Capsule (The same as Li 2009^12^). |
| Ou 2010^18^ | Lianhuaqingwen Capsule | The same as Li 2009^12^. |
| Qian 2010^19^ | Tanreqing Injection | The same as Han 2011^11^. |
| Qu 2010^20^ | Bingyanqing formula Ten | Radix Bupleuri 10g, Radix Puerariae 15g, Folium Isatidis 10g, Flos Chrysanthemi Indici 10g, Flos Lonicerae 10g, Radix Scutellariae 10g, Radix Saposhnikoviae 10g, Flos Magnoliae 10g, Rhizoma Belamcandae 10g, Radix Glycyrrhizae 5g. |
| Tan 2010^21^ | Modified Chaige Jieji Decoction; Yinhuang Granules; Shuanghuagnlian Oral Liquid | Modified Chaige Jieji Decoction (Radix Bupleuri, Radix Scutellariae, Radix Puerariae, Herba Schizonepetae, Flos Lonicerae, Folium Isatidis, etc. For patients with pain throat, Semen Oroxyli and Radix Sophorae Tonkinensis were added; for patients with cough and white sputum, Rhizoma Cynanchi Stauntonii and Rhizoma Pinelliae Preparatum were added; for patients with yellow sputum, Herba Houttuyniae was added). The compositions of Chinese herbs were not provided for Yinhuang Granules and Shuanghuagnlian Oral Liquid. |
| Tang 2010^22^ | Xiyanping Injection | Total ester sulfonate andrographolide. |
| Tian 2011^23^ | Qingkailing Oral Liquid | Calculus Bovis, Cornu Bubali, Radix Scutellariae, Flos Lonicerae, Fructus Gardeniae, Radix Isatidis, and etc. |
| Wang 2011^24^ | Maxingshigan-yinqiaosan | Honey-fried Herba Ephedrae 6g; Rhizoma Anemarrhenae 10g; Herba Artemisiae Annuae 15g; Gypsum Fibrosum 30g; Flos Lonicerae Japonicae 15g; Radix Scutellariae 15g; stir-baked Semen Armeniacae Amarum 15g; Fructus Forsythiae 15g; Fructus Forsythiae 6g; Bulbus Fritillariae Thunbergii 10g; Fructus Arctii Tosum 15g; and Radix Et Rhizoma Glycyrrhizae 10g. |
| Weng 2010^25^ | Qingjie Huashi Decoction | Flos Lonicerae 15g, Fructus Forsythiae 15g, Herba Menthae 5g, Periostracum Cicadae 5g, Rhizoma et Radix Notopterygii 10g, Radix Angelicae Pubescentis 10g, Herba Moslae 10g, Fructus Xanthii 10g, Radix Platycodonis 10g, Semen Armeniacae Amarum 10g, Raw Gypsum Fibrosum 30g, Baked Herba Ephedrae 3g, Radix Glycyrrhizae 6g, (for patients with exuberant heat and constipation, Radix et Rhizoma Rhei 6-10g was added；for patients with exuberant damp, Herba Artemisiae Scopariae 6-10g was added). |
| Ye 2010^26^ | Reduning Injection | Herba Artemisiae Annuae, Flos Lonicerae, Fructus Gardeniae. |
| Zeng 2011^27^ | Maxingshigan Decoction | honey-fried Herba Ephedrae 6g, Rhizoma Anemarrhenae 10g, Herba Artemisiae Annuae 15g, Gypsum Fibrosum 30g, Flos Lonicerae 15g, Radix Scutellariae 15g, stir-baked Semen Armeniacae Amarum 15g, Fructus Forsythiae 15g, Herba Menthae 6g, prepared Bulbus Fritillaria 10g, stir-baked Fructus Arctii 15g, Radix Glycyrrhizae10g. |
| Zhang 2011^28^ | Self-prescribed Chinese herbs | Flos Lonicerae 30g, Fructus Forsythiae 30g, Fructus Arctii 15g, Herba Menthae 15g, Herba Schizonepetae 15g, Lasiosphaera seu Calvatia 15g, Radix Scrophulariae 30g, Rhizoma et Radix Notopterygii 15g, Radix Gentianae Macrophyllae 15g, Radix Platycodonis 15g. |
| Zhao 2010^29^ | Qingwen Tuire Decoction | Flos Lonicerae 18g, Fructus Forsythiae 10g, Gypsum Fibrosum 30g, Rhizoma Anemarrhenae 10g, Radix Platycodonis10g, Folium Isatidis 15g, Radix Scrophulariae 12g, Radix Ophiopogonis 10g, Rhizoma Phragmitis 40g, Radix Scutellariae 10g, Rhizoma Belamcandae 9g, Radix Isatidis 15g, Radix Glycyrrhizae 9g. |
| Zhao 2011^30^ | Modified Sangju Decoction; Modified Chaige Jieji Decoction; Modified Puji Xiaodu Decoction; Huopu Xialing Decoction and Gegen Cenlian Decoction | Modified Sangju Decoction (Folium Mori 10g, Flos Chrysanthemi 6g, Radix Platycodonis 6g, Fructus Forsythiae 15g, Semen Armeniacae Amarum 10g, Herba Menthae 6g, Rhizoma Phragmitis 20g, Radix Scrophulariae 15g, Radix Isatidis 15g, Fructus Arctii 10g, honey-fried Radix Asteris 10g, Radix Glycyrrhizae6g); Modified Chaige Jieji Decoction (Radix Bupleuri 15g, Radix Puerariae 30g, Gypsum Fibrosum 30g, Radix Saposhnikoviae 10g, Flos Lonicerae 15g, Fructus Forsythiae 20g, Fructus Arctii 15g, Radix Scrophulariae 30g, Herba Menthae 6g, folia bambosae 10g, Rhizoma et Radix Notopterygii 15g, Radix Angelicae Dahuricae 10g, stir-baked Radix Scutellariae 10g); Modified Puji Xiaodu Decoction (Radix Scutellariae 15g, Gypsum Fibrosum 30g, Rhizoma Coptidis 15g, Radix Scrophulariae 30g, Fructus Forsythiae 15g, Lasiosphaera seu Calvatia 10g, Fructus Arctii 15g, Radix Platycodonis 6g, Bombyx Batryticatus 6g, prepared Rhizoma Cimicifugae 6g, Semen Coicis 30g, Herba Patriniae 10g, Rhizoma Phragmitis 10g, Semen Armeniacae Amarum 10g); Huopu Xialing Decoction and Gegen Cenlian Decoction (Agastache rugosus 10g, Rhizoma Pinelliae processed with ginger 10g, Semen Coicis 30g, Folium Perillae 10g, Rhizoma Atractylodis 10g, Radix Puerariae 20g, stir-baked Radix Scutellariae 10g, Rhizoma Coptidis 6g, Fructus Ammomi Rotundus 30g, Cortex Magnoliae Officinalis 10g, Massa Medicata Fermentata 10g) |
| Zheng 2010^31^ | Yinqiao Powder; Sanao Decoction | Yinqiao Powder (Flos Lonicerae, Fructus Forsythiae, and etc); Sanao Decoction (Radix Glehniae 15g, Radix Ephedrae 15g, Semen Armeniacae Amarum 10g, Radix Platycodonis 10g, Bulbus Fritillariae Thunbergii 15g, Herba Ardisiae japonicae 20g, Radix Stemonae 15g, Bombyx Batryticatus 10g, Rhizoma Belamcandae 10g, Radix Asteris 10g, Radix Glycyrrhizae 6g) |
| Zhu 2010^32^ | Gegen Granules | Radix Puerariae 12g, Herba Ephedrae 9g, Ramulus Cinnamomi 6g, Rhizoma Zingiberis Recens 9g, Radix Glycyrrhizae 6g, Paeonia lactiflora 6g, a dozen of Fructus Jujubae. |
